# Nano-microplastic and agro-ecosystems: a mini-review

**DOI:** 10.3389/fpls.2023.1283852

**Published:** 2023-11-20

**Authors:** Krishan K. Verma, Xiu-Peng Song, Lin Xu, Hai-Rong Huang, Qiang Liang, Chandra Shekhar Seth, Yang-Rui Li

**Affiliations:** ^1^ Sugarcane Research Institute, Guangxi Academy of Agricultural Sciences/Key Laboratory of Sugarcane Biotechnology and Genetic Improvement (Guangxi), Ministry of Agriculture and Rural Affairs/Guangxi Key Laboratory of Sugarcane Genetic Improvement, Nanning, Guangxi, China; ^2^ Department of Botany, University of Delhi, Delhi, India

**Keywords:** degradation of plastic, response of plants, envirotoxicology, soil contamination, micro(nano)plastics, pollution, remediation

## Abstract

Plastics’ unavoidable and rampant usage causes their trash to be extensively dispersed in the atmosphere and land due to its numerous characteristics. Because of extensive plastic usage and increased manufacturing, there is insufficient recycling and a large accumulation of microplastics (MPs) in the environment. In addition to their wide availability in the soil and atmosphere, micro- and nanoplastics are becoming contaminants worldwide. Agro-ecosystem functioning and plant development are being negatively impacted in several ways by the contamination of the environment and farmland soils with MPs (<5 mm) and nanoplastics (<1 µm). The contributions of some recyclable organic waste and plastic film mulching and plastic particle deposition in agroecosystems may be substantial; therefore, it is crucial to understand any potentially hazardous or undesirable impacts of these pollutants on agroecosystems. The dissolution of bioplastics into micro- and nano-particles (MBPs and NBPs) has not been considered in recent studies, which focus primarily on agro-ecosystems. It is essential to properly understand the distribution, concentration, fate, and main source of MPs, NPS, MBPs, and NBPs in agroecosystems. Based on the limited findings, understanding the knowledge gap of environmental impact from micro and nanoplastic in farming systems does not equate to the absence of such evidence. It reveals the considerations for addressing the gaps to effectively protect global food safety and security in the near future.

## 1 Introduction

Microplastics (MPs) have recently encouraged researchers to explore an emerging field of research. Plastics in the environment are degraded by photochemical, thermal, and biological processes (Fok et al., 2020). Plastics can be utilized for various applications, including electronic components, noise reduction, sealing, and insulation (Awasthi et al., 2017). Because of their wide range of applications, durability, hydrophobicity, low thermal and electrical conductivity, availability, and relatively low costs of manufacture, plastics are considered to be indispensable materials on a global scale, which has led to a continual increase in human demand for such materials (Adelodun, 2021; Iqbal et al., 2023). Smaller than 5 mm in size of plastic are generally referred to MPs. The majority of MPs are produced through large plastic break-offs. Additionally, MPs with a polymer composition similar to the plastic used for water transportation can be released into drinking water due to aging (Maddela et al., 2023) by adsorbing infectious agents, hazardous metals, and persistent organic pollutants (POPs) from the environment. Significant amounts of plastics are improperly disposed of on bare land, oceans, waterways, and drainage systems due to flagrant abuse of plastics and inadequate waste management. Flooding occurs when drainage systems become blocked and sewers overflow (Alabi et al., 2019).

MPs provide more ecological risks than their bulkier counterparts due to their small size, high specific surface area, and strong adsorption capability. MPs contamination increasing in the water and environmental protection research. However, the environmental factors controlling the emergence of MPs are poorly understood and explored. Sources of pollution, human activities, and hydrodynamic variables impact MPs accumulation and movement in ecological systems and food webs (Ng et al., 2018; Jia et al., 2023). If plastic pollution is not reduced, it will surpass and outweigh the Pisces on or before 2050, according to the World Economic Forum 2019 (Schwab, 2019). Humans in the modern era cannot live without plastics. Even if all future plastic production is prohibited, the ongoing problem will continue for an extended period because plastics have already contaminated each component of the environment (Godfrey, 2019; Jia et al., 2023).

The health of human beings, the environment, and aquatic life are susceptible to damage from microplastics (MPs; <5 mm) and nanoplastics (NPs; <1 µm), which are pervasive and can pose severe hazards (Kumar and Seth, 2021; Kumar et al., 2023; Okeke et al., 2023). Due to their small size, expected accessibility, direct and indirect intake of plastic particles, bioavailability, and increased concentrations of sorbed toxic chemicals, micro (nano) plastics (MNPs) are generally thought to pose more environmental and health risks (Yu and Chan, 2020; Roy et al., 2022; Mariyam et al., 2023). Microplastics exist in a variety of sizes and have been classified as primary or secondary microplastics based on whether they are micron-sized commercially produced plastics or smaller plastics that have been physically or chemically dispersed from larger plastics (macroplastics) (Jaikumar et al., 2019).

**Figure 1:**
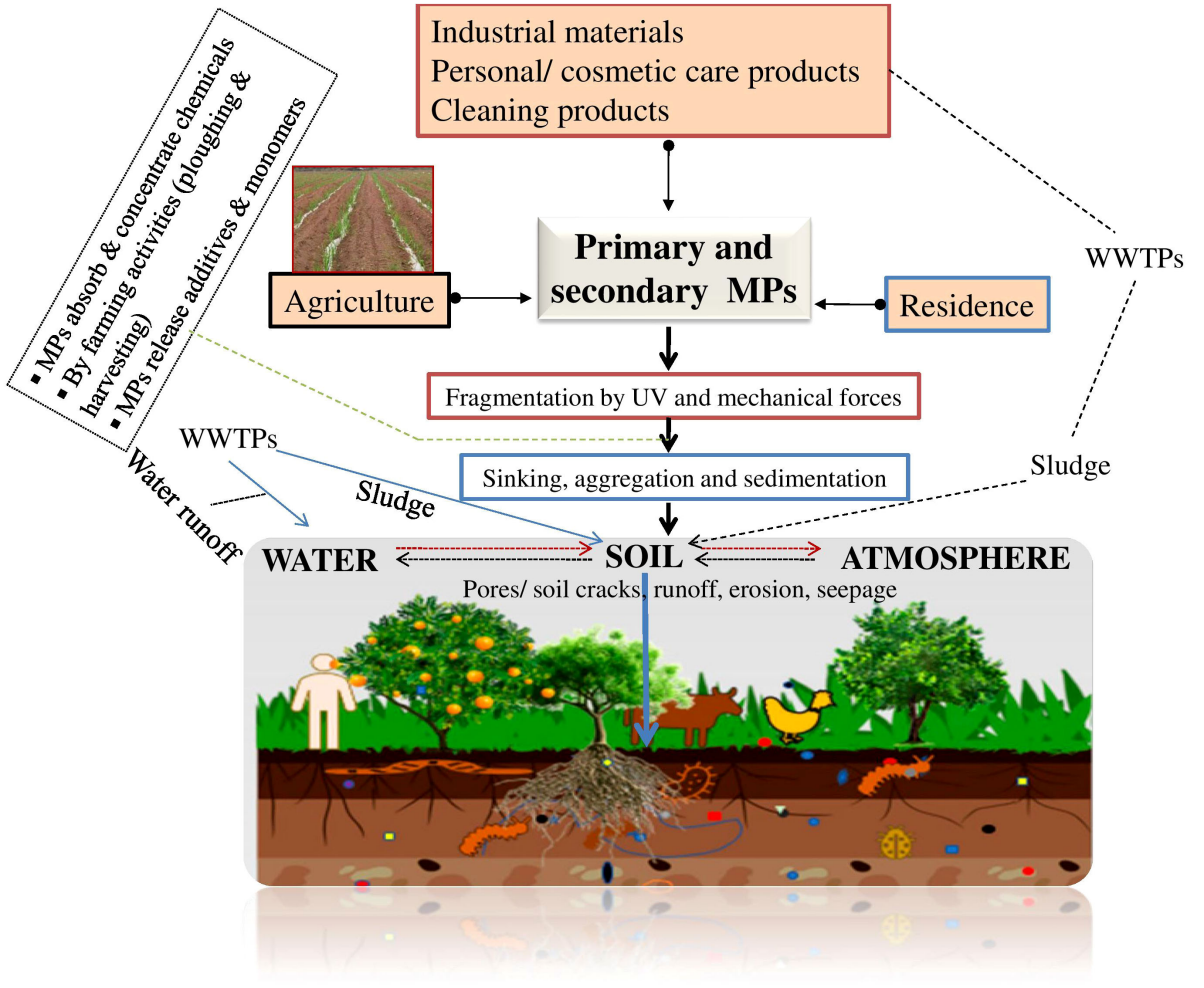
An overview of the sources of plastic wastes and transportation routes. WWTPs, wastewater treatment plants.

Primarily, MPs made for commercial uses include plastic pellets, microbeads, personal care items, and microfibers. Primary microplastics have also been used as pharmaceutical delivery systems in the healthcare sector. On the other hand, secondary microplastics (SMPs) are produced when plastics break down into smaller fragments after they have been released into the environment (Shafea et al., 2023) (**Figure 1**). Plastics are widely used in a variety of applications across the world as pervasive components of modern life. Almost 4.9 billion metric tonnes of plastic are produced annually, with around 60% of all plastics being thrown and accumulating in landfills and the environment. However, assuming current waste management patterns continue, 12 billion tonnes of plastic rubbish will enter landfills or the natural environment by 2050 (Geyer et al., 2017). Due to the purposeful use of plastic (such as plastic mulch, greenhouses, and products coated in plastic) as well as the use of sewage sludge, compost, and irrigation water that is contaminated with plastic, plastic pollution in agricultural soils has drawn increasing attention (Iqbal et al., 2023). This review discusses the possible environmental effects of micro (nano)plastics and how they might interact with biological contaminants.

## 2 Micro(nano)plastics: sources and distribution

Plastic contamination has become pervasive in farming land, posing a threat to food production systems, human health, and the environment. The essential sources of MNPs in agricultural ecosystems are the standard approaches, i.e., the application of biosolids (Koutnik et al., 2021) and compost, the use of plastic mulching films (Steinmetz et al., 2016; Corradini et al., 2019), water-pipes, cover of plastic greenhouse, polymer-based fertilizers, and pesticides (Weithmann et al., 2018; Corradini et al., 2019; Wang et al., 2019) (**Figure 1**). Aerial deposition and translocation from landfills are other considerable sources of MNPs in farmland soil (Zhang et al., 2018). The plastic film mulching approach is more advance among farming societies to protect soil moisture content, modulate soil temperature, and protect weed growth. Light-density polyethylene (LDPE) has been utilized to millions of hectares of farming lands worldwide (Yan et al., 2015). As the plastic film, Polyvinyl chloride (PVC) has enhanced the efficiency of water-use (WUE) and plant development and productivity. The “White Revolution” of plastic film mulch methodology is becoming “White contamination” in the agro-production systems. The dumping of municipal wastes in open lands, parks, or landfills has been a major factor in spreading MPs to soils in arid and semiarid regions (Kumar et al., 2020). About 40% of MNPs that reach farming soil cannot be recovered. These toxic contaminants break down into a continuum of smaller fragments (Wang et al., 2020).

Plastic contaminants can enter the agricultural production systems from damaged, degraded, or discarded agricultural plastic products and the leakage from non-agricultural sources like polluted water, air, and waste (Oliviero et al., 2019). Plastic trays and food contact films used for consumer packs for the distribution and retail to minimize the loss of food and conserve quality are also discarded in farming land (Sintim and Flury, 2017). Most agricultural plastics are single-use products; with short life spans, it becomes waste within one-year. The plastic mulching films decompose due to weathering, and the MPs that fall off from them remain in the soil. Improper disposal and mismanaged waste plastics will enhance soil contamination by SMPs. The abundance of MNPs may vary depends on the sampling locations and other related factors to usage of plastic and atmospheric conditions. The main polymers applied for the agri-systems are polyethylenes (PEs) of low- and high-density, polypropylene (PP), and PVC, followed by others, i.e., expanded polystyrene (PS), ethylene-vinyl acetate copolymer, and polyethylene terephthalate (PET) (Sarkar et al., 2018). The different kinds of polymers and the additives applied in agricultural plastics present a high degree of variability in the toxicological characteristics and the risks of MNPs to flora and fauna. The plastic movement begins from soil to aquatic environments via erosion and surface runoff at the end of each crop-growing season.

Because of the widespread use of plastic on agricultural land and pastures, secondary MPs are the primary source of plastic leftovers in farmlands (Corradini et al., 2021; Roy et al., 2022). The spatial modeling of farmland MPs demonstrated that agricultural techniques like crop management, i.e., fertilizers, organic amendments, irrigation, harvesting, and storage and cropping systems such as greenhouse crops or specific crops under plastic coverage play a significant role in MPs distribution into the rhizospheric soil. Accordingly, plastic can influence how particles, water, chemicals, and microorganisms interact in soil systems. The formation of MPs on cultivated lands can negatively impact the various responses of agroecosystems (Horton and Barnes, 2020; Mariano et al., 2021).

Yearly, the agricultural value chains and food packaging use nearly 12.5 Mt and 37.5 Mt of plastics (FAO, 2021). Plant and livestock production accounts for over 80% of plastic use, followed by fisheries and aquaculture (18%) and forestry (2%) in the agricultural value chains. The plastic protective films such as fumigation, silage, and bale wrap films, the protective films for mulching, nursery, wind tunnel, greenhouse, direct cover and non-woven floating cover, nets (ant-hail, anti-bird, wind-breaking, and shading), twine, and pipes for irrigation and drainage are broadly applied in agri-horticultural crops and livestock production. The breakdown process of plastics begins with the action of handling, soil abrasion, water, wind, and UV light. The breakdown products of varying sizes, including MNPs can last long in these production systems (Maddela et al., 2023).

## 3 Effects of MPs on soil health

**Figure 2:**
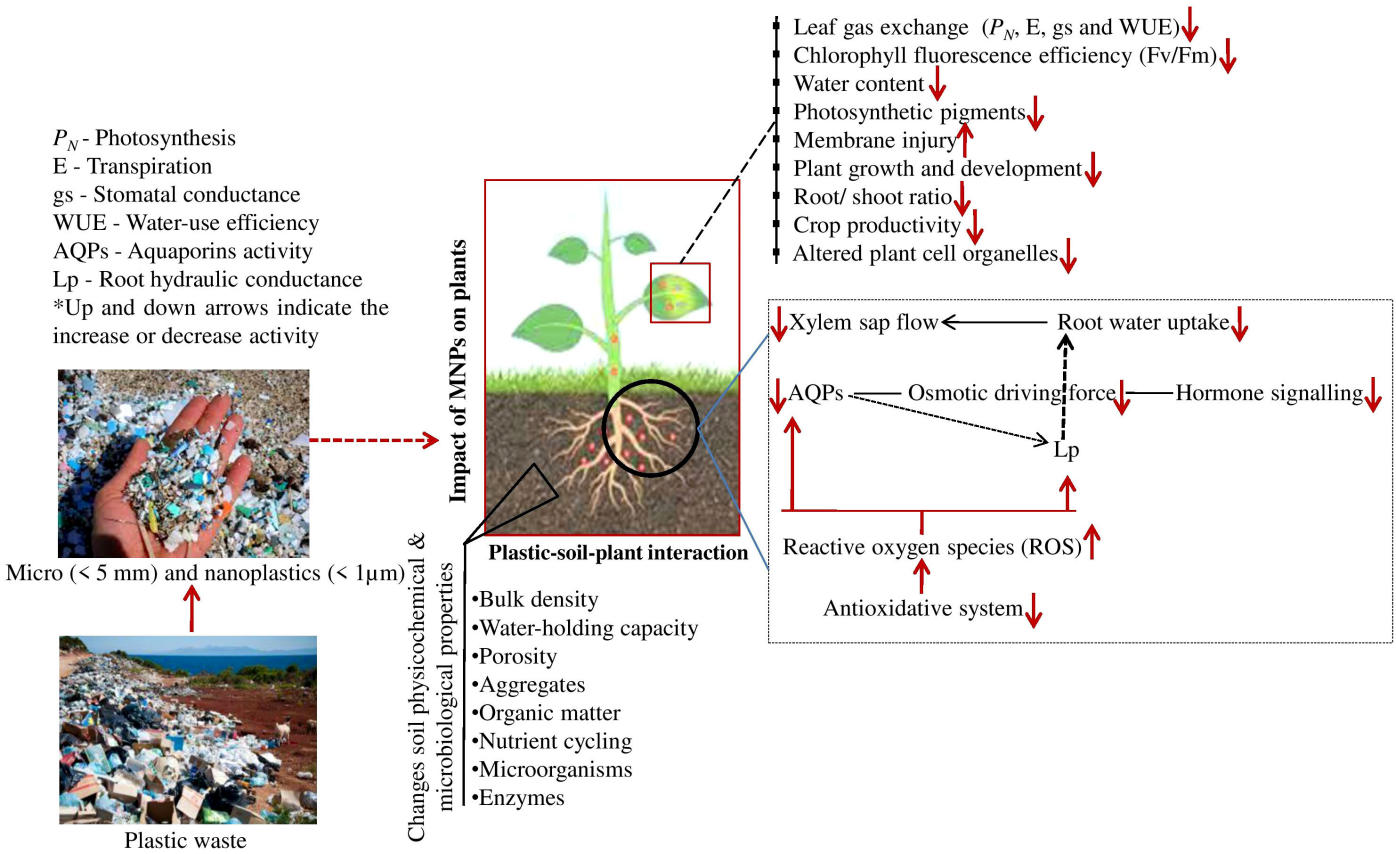
An overview of the micro-nanoplastics (MNPs) effects on plants via altering the soil physicochemical, microbiological properties, plant growth and development.

Soil structure becomes essential for edaphic conditions, soil fertility, water dynamics, and air permeability. According to research (Lian et al., 2021), the presence of MPs in soil may influence soil variables such as soil organic matter (SOM), pH, electrical conductivity (EC), and organic carbon storage. The degree of exposure and the number, kind, and size of the MPs all influence the outcome. According to Boots et al. (2019), soil pH decreased by up to 0.6 pH units compared to the control following one month of exposure to fibres added at 0.001% (w/w), high-density polyethylene (HDPE), and biodegradable polylactic acid (PLA), both added at 0.1% (w/w). After two months of exposure compared to the control, the application of MPs (low-density polypropylene and biodegradable materials, at 1% (w/w), caused an increase in soil pH; after four months of exposure, soil pH reduced again (Qi et al., 2019), that the exposure time is a controlling factor on pH-changes by MPs. Due to soil aeration and porosity, the pH of the soil can improve, which might cause the chemical additives in the MPs to leach into the ground and transform organic N into inorganic NH_4_^+^ (Zhao et al., 2021). However, structure, type, different MPs substances, enzymatic activities of the soil biota, and plant species significantly influence the pH of soil addressed by MPs. The field investigation on polymer-coated fertilizers’ impact on maize growth improved soil EC (Lian et al., 2021) (**Figure 2**).

Additionally, MPs may affect the soil C:N ratio. Two and four months after treatment, LDPE and biodegradable plastics significantly boosted the soil C:N ratio compared to the control (Qi et al., 2019). The size, type, and concentration of MPs have an impact on SOM concentration as well. Applying plastic mulch film in cotton cultivation areas in large diameters (0-200 cm^2^) and at concentrations of 250 to 2000 kg m^2^ has decreased SOM (Seth and Misra, 2014; Dong et al., 2015; Agnihotri and Seth, 2020). According to Boots et al. (2019), clothing fibres enhanced SOM, but biodegradable PLA and HDPE caused SOM to decrease relative to the control. It was discovered that the different plastic forms showed positive and negative priming effects on the SOM. The beneficial effect is caused by some plastic types degrading more rapidly with less persistence, which increases the C supply, microbial activity and proliferation, and exoenzyme activity. It may boost native SOM mineralization by metabolism (Zhou et al., 2020). The combined impacts of MP shape, size, type, and concentrations on soil organic carbon dynamics are currently unexplored. MPs effects on dissolved organic matter, particularly on soil organic carbon and nitrogen dynamics, investigations using long-term field trials with various combinations of size and concentration of MPs in agricultural soil at different latitudes and climates are required (Seth, 2014).

Depending on the type of bonding agents, such as hydrophobic vs. hydrophilic chemicals, MPs may have different effects on the stability of soil aggregates. In contrast, MPs are known to have hydrophobic surfaces, which reduces the strength of soil aggregates (de Souza MaChado et al., 2019). In the rhizosphere of treated soils with MPs and plants, concurrently, more water-stable aggregates have been observed (de Souza MaChado et al., 2019). However, Research is still needed to determine how different MPs types and shapes influence aggregate stability. Due to reduced mobility, MPs formation in aggregates can improve their adsorption in soil. In that process, the plastic structure is also essential.

Additionally, there have been reports that MPs impact soil water percolation. However, additional research is urgently required to ascertain the depth to which MPs will ultimately penetrate the soil. As much as 1 m of soil could contain MPs (Weber and Opp, 2020). According to O’Connor et al. (2019), leaching is essential in releasing MPs into groundwater. Plastic waste dispersion via biopores has been recognized as a possible route for groundwater pollution, although little is known about this phenomenon. The upward migration of plastic in agricultural soils towards aquifer systems, particularly for MPs, was established by He et al. (2018) in a process similar to the well-known migration of natural particles in colloids (such as insecticides). According to various conditions, plastic mobility in agricultural soils may differ (Yan et al., 2020). MPs have a resilient soil transport capability influenced by soil texture and retention period. Additionally, additional research into the upward migration of MPs, risk assessments for plant uptake, and an enormous rise in the volume of data on the geographical dependencies of plastic contamination can help to develop a better understanding of MPs-contaminated soils (Guo et al., 2020).

MPs have a variety of impacts on soil; these include changing the soil microclimate, the microbial community, and diversity in the ground. The severity of influence that various MP forms have on soil microorganisms varies. Both PE-MPs and PVC-MPs dramatically decrease the variety and complexity of microbial communities, with PE having a greater impact than PVC (Fei et al., 2020). The microbial populations are impacted differently by different soil MPs concentrations. When MPs concentrations are low, soil microbial activity declines; when concentrations are high, it boosts (Kumar et al., 2020). MPs influence soil microorganism change as exposure time increases. MPs boost the variety and richness of bacterial communities at the start of incubation but do the opposite later on (Ren et al., 2020). MPs do not significantly influence the variety and activity of microbial communities (Yu et al., 2022). PE-MPs did not considerably influence the diversity of the microbial communities in the soil, and the diversity index of the microbial communities on MPs was substantially lower than that of the soil. Variations in MPs, soil characteristics, and exposure time can be attributed to these unpredictable results.

According to Zhou et al. (2021a), MPs may act as novel biological properties for microorganisms that reside at the soil-plastic interface, such as microplastispheres. By developing microbial regions on their surfaces, MPs can enhance particular microbial communities and influence how plants and microorganisms interact (Yu et al., 2022). Bacterial communities were substantially more abundant in MPs than in the surroundings, and they are essential for ecological processes involving the C or S cycles (Xie et al., 2021). MPs enhance the number of microbes associated with self-degradation. In contrast to fast-growing vegetative microorganisms, oligotrophic microorganisms are more prevalent when PHBV-MPs appear (Zhou et al., 2021a). MPs may develop novel microbial niches that encourage the growth of particular microbial groups, which could have unexpected impacts on ecosystem processes.

MPs have two main effects on soil microbial populations. Changing the physicochemical properties of the soil influences the habitats of microorganisms, hence influencing the microbial population. Conversely, MPs provide alternate ecological homes for bacteria, whereas the release of synthetic plasticizers interferes with their healthy development and growth.

## 4 Effects of micro(nano)plastic(s) on plant growth and development

Increasingly prevalent toxic effects triggered by MPs on various physiological and biochemical processes in plants. These effects include inhibiting plant growth, altering root traits, reducing biomass, delaying and reducing fruit yield, interfering with photosynthesis, causing oxidative damage, and producing genotoxicity (Hernández-Arenas et al., 2021; Jia et al., 2023) (**Figure 2**). Due to their possible effects on the soil-plant system, plastics in agricultural soils are currently the reason. Farms employing plastic mulch (0.15 g kg^1^) demonstrated a yield reduction with increased plastic residue (Gao et al., 2019). However, it is impossible to distinguish between the effects of MaPs, MPs, and NPs. Depending on the size, structure, and type of polymer used to make the MPs, the impact of their waste on plant growth may differ (Ebere et al., 2019). According to de Souza MaChado et al. (2019), *Allium fistulosum* responded to several MPs, indicating that PS and polyester fibres, but not HDPE particles, significantly boosted the root biomass. However, in MPs treatments, *Triticum aestivum*L. and *Citrus aurantium* L. exhibited reduced plant height, shoot biomass, and leaf area (Boots et al., 2019; Verla et al., 2020). *Lepidium sativum* L. germination rate was considerably decreased after 8 hours of exposure to 50, 500, and 4800 nm NPs, with the negative effects of particle size expanding. After 24 hr of exposure, however, the impact of NPs and MPs on seed germination disappeared, and germination improved by nearly 100%, irrespective of the plastic size or exposure concentration (Bosker et al., 2019).

According to reports, plastic waste influences soil’s physical, chemical, and biological characteristics, indirectly impacting plant growth. According to Qi et al. (2019), compared to LDPE debris, biodegradable plastic debris exhibited a greater influence on the number of microbes in the wheat rhizosphere and was associated with decreased plant biomass. Biodegradable residues can reduce plant biomass by releasing harmful chemicals into the soil solution and disrupting the microbiome (Ng et al., 2018). NPs may penetrate plants through the soil and build up in cells and tissues (Urbina et al., 2020). Under 5 μm, PS-MPs found that the biomass and catalase enzyme activity of *V. faba* roots decreased while the peroxidase and superoxide dismutase enzyme activity significantly increased (Jiang et al., 2019). At 100 mg L^-1^, significant growth reduction was observed. According to the surface charge of the NPs, Sun et al. (2020) demonstrated that positively and negatively charged NPs might accumulate in *Arabidopsis thaliana* with different results.

Positively charged NPs tended to concentrate due to the growth medium and root exudates, reducing their absorption compared to negatively charged NPs. However, compared to negatively charged NPs, positively charged NPs caused a greater buildup of reactive oxygen species (ROS) and hampered seedling and plant development. In contrast, negatively charged NPs were generally found in the apoplast and xylem. The negative effects of NPs on *Zea mays* L. on molecular photosynthesis were observed by Sun et al. (2022) through the reduction of photosystem II efficiency due to the downregulation of the transporter D1 protein, which has more pronounced inhibitory effect on plant growth and development (Wang et al., 2021).

According to Azeem et al. (2021); Sun et al. (2021), and other researchers, nanoparticles (NPs) significantly affect biochemical enzymes, the antioxidant system, electrolyte leakage, block cell wall pores, and trigger oxidative damage in plants. Although MPs have impacted growth parameters, it is presently unknown how MPs might change how plants function. The immobilization of nutrients by organic molecules is released during degradation (Jia et al., 2023). MPs can influence plant growth and performance through various approaches, including direct toxicity to plants and indirect effects on plant development through changes in soil characteristics and microbial populations (Rillig et al., 2019).

### 4.1 Direct responses of MPs on plants

The adsorption of MPs can influence plants’ performance. It might bind to plant roots and affect their characteristics, making it difficult for plants to absorb water and nutrients. According to Jiang et al. (2019), MPs with small particle sizes are more detrimental to plants. By modifying the state of cell membranes and intracellular molecules and generating oxidative stress, micro(nano)plastics at submicrometer or micron levels can penetrate and damage the plant body (Yin et al., 2021; Liu et al., 2022). According to Li et al. (2021a) and Liu et al. (2022), nano- and micro-sized particles may build up in the interspace tissues of plant roots before migrating to the leaves, stems, flowers, and fruits. The capacity of plants to absorb minerals like Fe, Mn, Cu, and Zn can also be affected by MPs, and the phytotoxicity of MPs varies unquestionably depending on different plant species or cultivars (Gong et al., 2021).

MNPs could upregulate ROS production in various crops, suggesting that MNPs could induce oxidative damage in the crops (Zhou et al., 2021b; Chang et al., 2022; Colzi et al., 2022) (**Figure 2**). Enhanced ROS could diminish the production of amino acids, nucleic acids, lipids, and other secondary metabolites. The current study showed that excessive ROS generation beyond the antioxidant system’s scavenging capabilities weakened membrane functions (Wu et al., 2020). Specifically, MNPs could interfere with metabolic pathways of carbohydrate, amino acid, alanine, aspartate, and glutamate in plants and change the regularity of these pathways by promoting or inhibiting related gene expression, thereby upgrading crop adaptation strategy to MNPs stressors and influencing growth and development of plants (Wu et al., 2022; Zeb et al., 2022; Zhang et al., 2022). MNPs-induced gene transcription in plants could regulate the stimulation or inhibition of plant hormones, cell proliferation, and uptake of nutrients (Wu et al., 2022). However, MNPs could contribute to significant imbalances in galactose, pentose phosphate, starch, and sucrose metabolisms in specific crops. Plant metabolisms are modulated by plant hormones, which can regulate enzymatic activities in response to MNPs stresses (Li et al., 2021b). Therefore, variations in metabolic processes may interact with the availability of nutrients, antioxidative defense systems, energy production, and biosynthetic pathways, thus potentially inhibiting growth, development, and crop productivity (**Figure 2**).

### 4.2 Indirect effects of MPs on plants through changes in rhizospheric soil

The biological community, variety, and soil qualities all have an important role in plant growth. MPs impact the physicochemical properties and microbial populations of the soil, which can modify the rhizosphere, plant development, and nutrient availability, hence harming plants (**Figure 2**). MPs dramatically increase the rate of soil water evaporation, which can cause soil dryness and plant performance problems (Wan et al., 2019; Amobonye et al., 2021). MPs may decrease soil fertility and result in nutrient loss for plants. Additionally, MPs can reduce the diversity of soil microbes or the quantity of rhizosphere fungal symbionts, which may reduce plant diversity (Bandini et al., 2022).

## 5 Mitigation strategies

Efforts from various steps are being devoted to attenuating the harmful effects of plastic fragments arising from the current large-scale enhancement in the production of plastic and its use. Assessing the impacts of MNPs in the agro-environment on an economic scale is inherently difficult because of the knowledge gaps. MPs/NPs remediation or solution to this challenge in cropping land, the first concern is from where the problem is originating. Sources of plastic mulch contribute considerable to MPs/NPs pollution. Organic mulching materials instead of plastic ones, i.e., residue of crops, tree leaves, rice straws, husks, wood dust, and water hyacinths are promoted in conservation agriculture because it decompose easily (Mancinelli et al., 2015). These materials may be difficult to manage at times and can be problematic for the coverage of large area. As a result, moving from plastic mulches to organic and biodegradable sources can be a beneficial strategy for minimizing the threats of MPs/NPs contamination to maintain environmental sustainability. For instance, a fully biodegradable polymer and natural fiber based on starch could be a better way to minimize the huge amount of plastic waste in agricultural fields (Tan et al., 2016). Natural polymers can also be applied to substitute plastic polymer-coated fertilizers. By 2026, 59% of plastic mulch will be consumed worldwide in sustainable agriculture (starting in 2018) (Sintim et al., 2020). Biodegradable films dissociate in the soil for variable lengths of time depends on the atmospheric variables that can be related to the degradation rate of compost is observed to be faster than soil (Sintim et al., 2020). Similarly, depending on the components and temperature of pyrolysis, biochar application in MPs/NPs-contaminated soil can enhance soil nutritional profile (Palansooriya et al., 2022; Roy et al., 2023).

The use of biosolids is another approach for MPs/NPs to get into the soil. According to Peng and Pivato (2019), among the organic solids include sewage sludge, cattle manure, kitchen/food waste, and agricultural byproducts, all of which have significant amount of MPs/NPs, heavy metals, and other organic contaminants. Hence, before applied to farmland such materials should be applied for two reasons. The main purpose is to eliminate MPs/NPs, and the secondary objective is to minimize the heavy metals toxicity or other dangerous substances. Currently, researchers have focused on the development of modern approaches to process sustainable biosolids. For instance, hyperthermophilic composting (HTC) is more successful than other conventional solid waste treatment applications regarding MPs/NPs and heavy metal contamination reduction (Chen et al., 2021),. The several bacteria have thermophilic characteristic that facilitate biodegradation. Biodegradable plastics are regarded as safer than synthetic plastics and more prone to microorganisms (Bhatt et al., 2021; Liao and Chen, 2021); thus, adopting biodegradable plastics and microbial degradation of plastic waste would be a potential remedy to MP/NP pollution. In addition, it is also argued that biodegradable microbeads (chito-beads) used in cosmetics exhibited greater cleansing efficiency than polyethylene (PE) microbeads and completely degraded in soil into CO_2_, H_2_O and biomass without any toxic effects on plants. Consequently, the development of biodegradable plastics and engineered microorganisms which can easily convert plastic particles either from conventional plastics or biodegradable plastics and mineralize them would be the key to MP/NP remediation; thus, they could be environmentally benign (Ju et al., 2021; Roy et al., 2022).

Encapsulated silica has been successfully used to eliminate heavy metal and hydrocarbon pollution of water and soil. Therefore, it is potential that encapsulated enzyme treatment could be effective in reducing MPs and NPs from the soil. The use of encapsulated enzymes, mainly near/around seeds during sowing or even close to the plant root zone, can develop a modern era of MPs/NPs restoration from the farmland soil. As a result, the enzymes will be produced from the capsule near the germinating seeds that can protect them from the adhering of MPs/NPs. It will help to degrade MPs/NPs that have bound to the plant roots (Camenzuli et al., 2017). Melatonin application may be an alternative approach for controlling MPs/NPs contamination in soil and plants. Melatonin enhanced plant tolerance efficiency to MPs/NPs toxicity, and stimulates ROS scavenging to improve redox homeostasis. The frequency of MPs/NPs contamination by air deposition is high in metropolitan and suburban areas, particularly along roads and in industrial locations. Therefore, it is recommended to plantation a variety of trees at various heights near to roadsides and industrial locations. It will act as windbreak and air screening. An outcome, air deposition of MPs/NPs and other contaminants to crop plants can be minimized. After a certain time, the border trees may be utilized to make furniture and green energy sources (Roy et al., 2023).

## 6 Conclusion and future perspectives

The harmful impacts of soil and ecosystem contamination on plant growth and development are a serious issue that must be addressed urgently. To limit pollutant discharge into agroecosystems, it is critical to prioritize reducing plastic waste, improving waste management systems, supporting sustainable practices, and enacting legislation. Because of the growing use of plastic-based items and incorrect disposal, farmland has become a key pollution sink for various plastic wastes and MPs. Because of their durability and resistance to degradation, MPs are abundant in agricultural soils, which can be hazardous to the ecology. Specific mitigation and management approaches to MP contamination in agroecosystems are still lacking. However, it is widely accepted that the development of biodegradable plastic products as alternatives, the “Plastic Restriction Order” that restricts the use of first-generation MPs and plastic products, the recycling and proper disposal of plastic waste, as well as the removal of plastic waste stocks, have positive effects on managing the source of MPs and on severing their route to being transported and accumulating in agricultural soils. The key preventative and control strategies are implementing control regulations, developing and using biodegradable plastics, and regulating plastic waste recycling and disposal. To effectively restrict the movement of MPs into the agroecosystems, the public, business, and government need cooperation for future betterment.

## Author contributions

KV: Data curation, Formal analysis, Resources, Software, Writing – original draft. XS: Data curation, Funding acquisition, Resources, Software, Supervision, Validation, Writing – original draft. LX: Data curation, Funding acquisition, Resources, Software, Writing – review & editing. HH: Data curation, Resources, Software, Writing – review & editing. QL: Funding acquisition, Resources, Software, Writing – review & editing. CS: Formal analysis, Resources, Software, Supervision, Writing – review & editing. YL: Conceptualization, Funding acquisition, Investigation, Project administration, Software, Supervision, Validation, Visualization, Writing – review & editing.
